# Neural repetition suppression: evidence for perceptual expectation in object-selective regions

**DOI:** 10.3389/fnhum.2014.00225

**Published:** 2014-04-17

**Authors:** Lisa Mayrhauser, Jürgen Bergmann, Julia Crone, Martin Kronbichler

**Affiliations:** ^1^Centre for Neurocognitive Research, University of SalzburgSalzburg, Austria; ^2^Neuroscience Institute, Christian-Doppler Clinic, Paracelsus Medical UniversitySalzburg, Austria

**Keywords:** fMRI, lateral occipital complex, object processing, predictive coding, repetition suppression

## Abstract

It is an established finding that neuronal activity is decreased for repeated stimuli. Recent studies revealed that repetition suppression (RS) effects are altered by manipulating the probability with which stimuli are repeated. RS for faces is more pronounced when the probability of repetition is high than when it is low. This response pattern is interpreted with reference to the predictive coding (PC) account, which assumes that RS is influenced by top-down expectations. Recent findings challenge the generality of PC accounts of RS by showing repetition probability does not modulate RS for other visual stimuli than faces. However, a number of findings on visual processing are in line with PC. Thus, the influence of repetition probability on RS effects during object processing requires careful reinvestigations. In the present fMRI study, object pictures were presented in a high (75%) or low (25%) repetition probability context. We found increased RS in the high-probability context compared to the low-probability context in the left lateral occipital complex (LOC). The dorsal-caudal and the ventral-anterior subdivisions of the LOC revealed similar neuronal responses. These results indicate that repetition probability effects can be found for other visual objects than faces and provide evidence in favor of the PC account.

## Introduction

Repetition suppression (RS) is commonly defined as a diminished neural activation that results from the repeated presentation of a stimulus (Henson, 2003). In functional magnetic resonance imaging (fMRI), this pattern becomes manifest as the reduced blood oxygen-level-dependent (BOLD) response elicited by a repeated stimulus, also called fMRI adaptation (Grill-Spector and Malach, 2001; for a recent review see also Segaert et al., 2013). Critically, its underlying neuronal mechanisms have been discussed as either arising from *neuronal fatique* (Grill-Spector et al., 2006), *neuronal sharpening* (Martens and Gruber, 2012), or *neuronal facilitation* (Grill-Spector et al., 2006), according to which RS is a relatively automatic result of bottom-up mechanisms.

By contrast, the predictive coding (PC) model emphasizes the role of top-down influences on RS. According to this approach, information about a stimulus (e.g., an object) flows in a hierarchical manner from lower to higher cortical layers while expectations, which are built upon prior object regularities, are top-down backward influences which modulate the processing of the current object (Friston, 2005). Bottom-up flow of information and top-down expectations are then compared at each level of hierarchical processing. From this perspective, RS occurs due to a correct prediction of the upcoming stimulus, that is, the currently processed object matches the expectation. Therefore, RS effects reflect a smaller prediction error for expected stimuli, that is, decreased activation for repeated stimuli.

This model was recently put forward by the finding that a manipulation of expectations alters RS effects (Summerfield et al., 2008, 2011). In these studies, the probability with which pictures of faces were repeated altered between blocks, thus inducing a relatively high expectation of repetitions in high-probability blocks (75% repetitions) and a rather weak expectation of repetitions in low-probability blocks (25% repetitions). RS effects were more pronounced when repetition was expected compared to when it was less expected. These perceptual expectation effects cannot be explained by bottom-up mechanisms alone.

Findings challenging the PC account of RS revealed that RS effects during object processing in monkey inferior temporal cortex and human lateral occipital complex (LOC) were not modulated by repetition probability(Kaliukhovich and Vogels, 2011; Kovács et al., 2013; respectively), thus questioning the generality of top-down effects in visual perception. However, the notion that perceptual expectations about objects should not affect RS effects would be surprising, since prior investigations indicate that top-down effects modulate the processing of visual stimuli at each level of hierarchical processing. To illustrate, the results of Cardin et al. (2011) suggest that learnt regularities, which are related to increased activation in anterior visual and frontal areas, generate top-down signals. Additional support for the validity of PC in object perception is provided by recent findings indicating that RS is driven by changes in top-down effects in body-sensitive networks (Ewbank et al., 2011) and that top-down expectations and surprise effects account for neuronal responses in the FFA and PPA while pictures of faces and houses were processed (Egner et al., 2010).

Resolving the ambiguity whether RS during object perception is modulated by top-down effects is critical for a validation of the PC account beyond face processing. To address this, we investigated whether RS effects are modulated by repetition probability during object processing. Similar to prior studies of Summerfield et al. (2008, 2011) we performed an fMRI experiment where participants were presented with pictures of objects in either a high- or low-repetition probability context. Compared to the prior investigation of Kovács et al. (2013), who failed to detect RS modulation during object processing in humans, we aim to strengthen the contrast between high- and low expectation contexts by raising the probability with which pictures are repeated in the high-probability context to a higher level (75% repetitions compared to 60% in the study of Kovács et al., 2013). Furthermore, we assess RS effects in a larger sample in order to increase the chances to detect effects of repetition probability.

According to the PC approach, we expect that repeated stimuli in the high-probability context elicit a pronounced decrease in activation in the LOC, since the correct prediction of a repeated object facilitates its processing. Repeated objects are less expected in the low-probability context and thus RS effects should be less pronounced. To test the additional assumption of PC that the influence of object expectations is restricted to object-selective regions, we investigate RS effects in the FFA and the Parahippocampal Place Area (PPA), which are relatively dedicated to the processing of face and place stimuli, respectively. If this is the case, we expect RS effects not to be modulated by repetition probability in these regions. Although some accounts assume that visual representations are widely distributed in the visual pathway (e.g., Haxby et al., 2001), many studies show that some visual regions (including the FFA and the PPA) respond much stronger to one specific stimulus category compared to most other visual stimuli (Epstein et al., 1999; Kanwisher and Yovel, 2006).

## Methods

### Participants

Nineteen undergraduate students (11 female) from the University of Salzburg (age 18–30 years) participated in the present study. All participants had normal or corrected-to-normal vision and reported no history of neurological or psychiatric disease. They provided written informed consent and were remunerated for participation with structural images of their brains on CD and payment. All methods conform to the Code of Ethics of the World Medical Association (Declaration of Helsinki). The institutional guidelines of the University of Salzburg (Statutes of the University of Salzburg—see https://online.uni-salzburg.at/plus_online/wbMitteilungsblaetter.display?pNr=98160) state in § 163 (1) that ethical approval is necessary for research on human subjects if it affects the physical or psychological integrity, the right for privacy or other important rights or interests of the subjects or their dependents. In § 163 (2) it is stated that it is the responsibility of the PI to decide, whether (1) applies to a study or not. Therefore, we did not seek ethical approval for this study. Since it was non-invasive and performed on healthy adult volunteers who gave their informed consent to participate, (1) did not apply. Data was processed in anonymized/deidentified form. Upon arrival at the lab, participants were assigned a subject ID (v001, v002, etc.) which was used throughout the study.

### Stimuli and design

**Figure 1:**
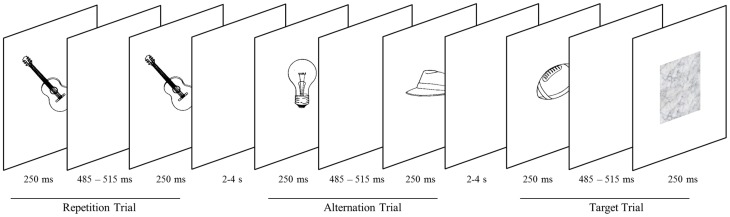
**Objects are presented in successive pairs separated by a blank screen**. Pairs either comprise the same object (repetition trials) or two different objects (alternation trial). Subjects screen stimuli for blurred objects (target trials), occurring on 20% of trials. Targets are presented in either the high-probability (75% probability of stimulus repetition) or the low-probability block (25% probability of stimulus repetition).

The majority of stimuli (examples are depicted in Figure 1) were monochrome object line-drawings from a standardized corpus (Szekely et al., 2004). Additional pictures downloaded from the public domain of the World Wide Web were matched in size and luminance to the pictures of the corpus. Since we were specifically interested in whether objects reveal perceptual expectation effects, the stimulus material does not include any scene and people drawings.

Stimuli were presented centrally on a white background through the scanner bore onto a mirror (at a distance of approximately 80 cm), which reflected the image to the participant. Two consecutive scan sessions consisted of 12 epochs each. Within a session, epochs alternated between high and low probability context. Runs were initiated and closed by a screen depicting the words “start” and “end,” respectively. Both screens were presented for 800 ms. The time lag between epochs was 1200 ms. Within an epoch, stimuli were presented in 20 successive pairs of pictures and each participant attended 480 pairs in total. Both the first and the second picture were presented for 250 ms (Figure 1). They were separated by a blank screen for a jittered time interval of 485–515 ms within a pair and a jittered inter-stimulus interval of 2000–4000 ms between pairs. Similar to prior investigations on RS (e.g., Summerfield et al., 2008), each stimulus pair was treated as compound trial. In epochs with a low probability of an image being repeated within a pair (low-probability context), images were either the same (25% of trials) or different (75% of trials). In the high-probability context, images were the same in 75% of trials. Participants were not explicitly told that the repetition probability was manipulated across epochs. To maintain their attention, participants had to indicate (via button box) blurred pictures which occurred on 20% of trials. Target pictures occurred equally often as first or second stimulus and were excluded from analyses.

### Localizer task

Subsequent to the main task, 16 out of 19 subjects performed a standard localizer task to define the LOC, the FFA, and the PPA. During the localizer task, participants passively viewed pictures of objects, faces, buildings, words, and scrambled objects. Each stimulus category was presented in six separate blocks (6 pictures per block resulting in 36 pictures for each condition). Each picture was presented for 800 ms, followed by a jittered inter-stimulus interval (1770–1830 ms) displaying a blank screen. Importantly, stimuli differed from those used in the main task.

### Image acquisition and data analysis

Functional imaging data were acquired with a Siemens Magnetom Trio 3 Tesla scanner (Siemens AG, Erlangen, Germany) equipped with a 12-channel head-coil. Functional images sensitive to BOLD contrast were acquired with a T2^*^ weighted gradient echo EPI sequence (TR 2000 ms, TE 30 ms, matrix 64 × 64 mm, FOV 192 mm, flip angle 70°). Thirty-six slices with a slice thickness of 3 mm and a slice gap of 0.3 mm were acquired within the TR. Scanning proceeded in two sessions with 526 scans per session. Six dummy scans were acquired at the beginning of each functional run before stimulus presentation started. Additionally, a gradient echo field map (TR 488 ms, TE 1 = 4.49 ms, TE 2 = 6.95 ms) and a high resolution (1 × 1 × 1.2 mm) structural scan with a T1 weighted MPRAGE sequence were acquired from each participant.

For preprocessing and statistical analysis, SPM8 software (http://www.fil.ion.ucl.ac.uk/spm/), running in a MATLAB 7.6 environment (Mathworks Inc., Natick MA, USA), was used. Functional images were realigned, unwarped and corrected for geometric distortions using the fieldmap of each participant and slice time corrected. The high resolution structural T1weighted image of each participant was processed and normalized with the VBM8 toolbox (http://dbm.neuro.uni-jena.de/vbm8) using default settings, each structural image was segmented into gray matter, white matter and CSF and denoised, then each image was warped into MNI space by registering it to the DARTEL template provided by the VBM8 toolbox via the high-dimensional DARTEL (Ashburner, 2007) registration algorithm. Based on these steps, a skull stripped version of each image in native space was created.

To normalize functional images into MNI space, the functional images were coregistered to the skull stripped structural image and the parameters from the DARTEL registration were used to warp the functional images, which were resampled to 3 × 3 × 3 mm voxels and smoothed with a 6 mm FWHM Gaussian kernel.

Statistical analysis was performed with GLM two staged mixed effects model. In the subject-specific first level model, each condition was modeled by convolving box-car functions (duration = 1 s) at its onsets (i.e., start of each compound trial) with SPM8′s canonical hemodynamic response function (as in Summerfield et al., 2008). Target trials and start and end messages were modeled as separate events of no interest, the model also included the six motion parameters as regressors of no interest. Parameter estimates for each condition were calculated via these first level general linear models (GLM), using a temporal high-pass filter (cut-off 128 s) to remove low-frequency drifts and modeling temporal autocorrelation across scans with an AR (1) process (Friston et al., 2002). For the voxel-based group analyses contrasts for effects were calculated at the first level and used for second level analyses using one-sample *t*-tests for each effect of interest. A threshold of *p* = 0.001 (uncorrected) with a minimum extent of 10 voxels was used for these exploratory group analyses.

### ROI analysis

The following Regions of interest (ROIs) were defined based on voxel-based group analyses of the localizer task by using specific *t*-contrasts: the LOC (object drawings > scrambled stimuli), the FFA (faces > scrambled stimuli) and the PPA (buildings > scrambled stimuli). To define the LOC, the PPA, and the right FFA, we used a voxel-level threshold of *p* < 0.001 (uncorrected) and a FWE correction of *p* < 0.05 for cluster extent. Since no FFA in the left hemisphere could be defined with this corrected cluster threshold, we used a liberal voxel-level threshold of *p* < 0.01, uncorrected. To facilitate the comparison of results to the data of Kovács et al. (2013), we split the LOC in two smaller ROIs [i.e., the lateral occipital (LO) and the posterior fusiform (PFs) region] according to the following criteria: all LOC voxels anterior to *y* = −63 and inferior to *z* = −13 were assigned to the PFs ROI, the remaining voxels formed the LO ROI (see Sayres and Grill-Spector, 2006).

These definitions led to the following locations in MNI space and sizes for the ROIs: left LOC −42, −76, −2 (331 voxels) including the left LO −33, −82, −11 (303 voxels) and the left PFs −42, −49, −17 (28 voxels), the right LOC 45, −82, −5 (308 voxels) including right LO 39, −73, −14 (286 voxels) and right PFs 39, −61, −11 (22 voxels), left PPA −24, −46, −14 (130 voxels), right PPA 27, −46, −20 (239 voxels), left FFA −33, −49, −26 (19 voxels) and right FFA 39, −55, −20 (176 voxels). The ROIs for the FFA and PPA are depicted in Supplementary Figures A.1 and A.2, respectively.

The coordinates of these ROI closely correspond to the location of these regions in previous studies (e.g., Kanwisher et al., 1997; Sayres and Grill-Spector, 2006; Baldassano et al., 2013).

## Results

### Behavioral results

Since RS is known to be modulated by attention (Larsson and Smith, 2012) we assessed whether behavioral results indicate possible attentional differences between the two probability contexts. In general, hit rates were at ceiling and participants responded correctly in more than 95% of trials. *T*-tests revealed that neither RT nor hit rates differed significantly between the high- and the low-probability context (*t* < 1.28, *p* > 0.2). Thus, behavioral data did not reveal apparent differences between the conditions.

### Imaging results

#### LOC

**Figure 2:**
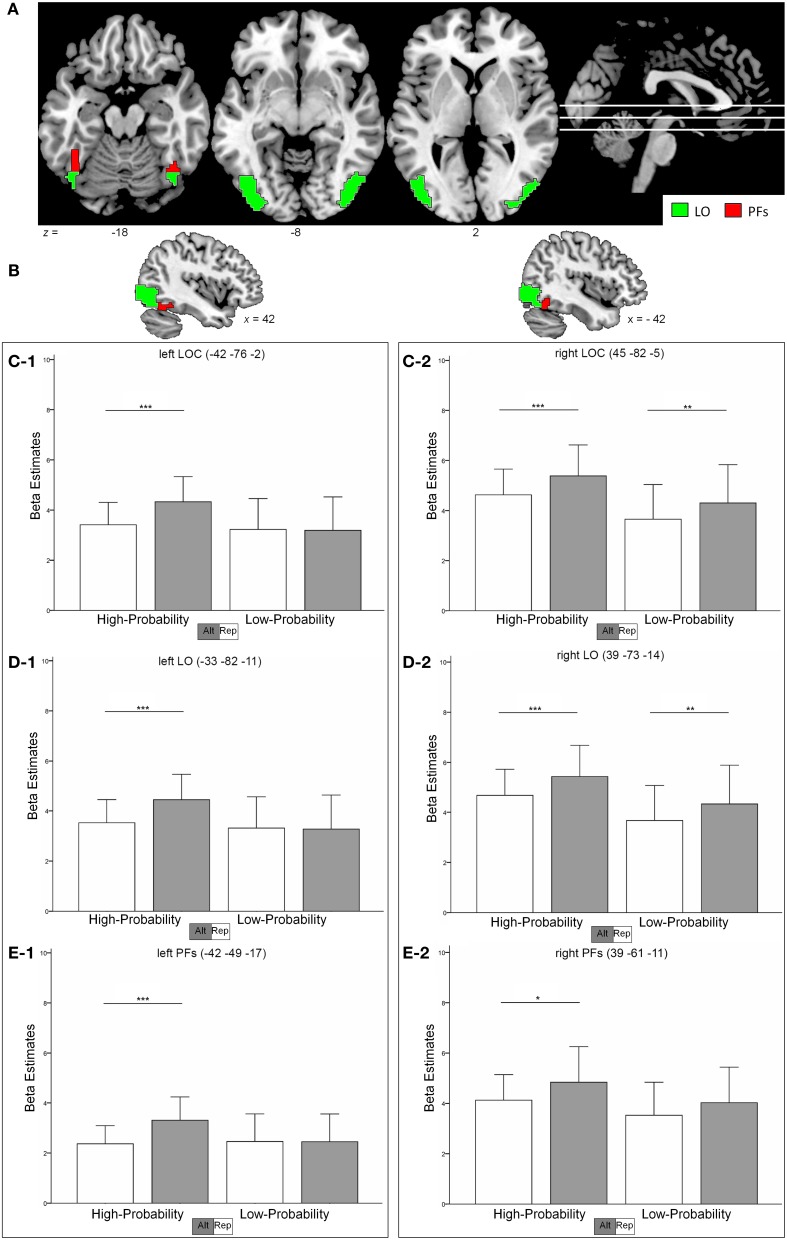
**(A,B)** shows the location of the LOC (sum of red and green clusters) as identified in the functional localizer. The subregions LO and PFs are depicted as green and red regions, respectively. **(C–E)**, Contrast estimates in the left and right hemisphere in the LOC **(C-1**, **C-2)** the LO **(D-1**, **D-2)** and the PFs **(E-1**, **E-2)**. Error bars indicate two standard deviations of the mean. ^*^p < 0.05; ^**^p < 0.01; ^***^p < 0.001 (*post-hoc* comparisons).

In the left LOC (Figure 2C1), strong RS effects could be observed in the high-probability context with a decrease of 21% for repeated as compared to alternate stimuli (*t* = 6.19, *p* < 0.001). However, no RS effects could be found in the low-probability context (1% activation increase for repeated trials; *t* = 0.246, *p* = 0.808). Formally, we found a main effect of stimulus type, with repeated trials eliciting less activation than alternate trials [*F*_(1,18)_ = 15.45, *p* = 0.001]. Critically, this main effect was qualified by a stimulus-by-probability interaction [*F*_(1, 18)_ = 21.68, *p* < 0.001].

To the contrary, RS effects in the right LOC (Figure 2C2) were similar in the high- and low-probability context with 15 and 14% (*t* > 2.87, *p* < 0.01) decrease in activation, respectively. The data revealed a main effect of stimulus type [repeated < alternate, *F*_(1, 18)_ = 20.97, *p* < 0.001] and of probability [high > low, *F*_(1, 18)_ = 5.53, *p* = 0.03]. However, the stimulus-by-probability interaction was not significant [*F*_(1, 18)_ = 0.148, *p* = 0.705].

An overall analysis (including hemisphere as an additional factor) revealed a significant three-way interaction between the factors hemisphere, probability, and stimulus type, indicating that repetition probability modulated RS effects differently in the left and right LOC [*F*_(1, 18)_ = 14.76, *p* = 0.001].

#### LO

Similar to the LOC, the activation pattern in the left LO (Figure 2D1) revealed a more pronounced RS effect in the high-probability context (21% signal decrease, *t* = 6.09, *p* < 0.001) as compared to the low-probability context (1% signal increase, *t* = 0.256, *p* = 0.801). Again, the data were characterized by a stimulus-by-probability interaction [*F*_(1, 18)_ = 19.77, *p* < 0.001] and a main effect of stimulus type [repeated < alternate, *F*_(1, 18)_ = 15.05, *p* = 0.001].

The right LO (Figure 2D2) revealed similar RS effects in both contexts [15 and 14% decrease in the high- and low-probability context, respectively (*t* > 2.96, *p* < 0.008)] and thus no significant stimulus-by probability interaction could be observed [*F*_(1, 18)_ = 0.129, *p* = 0.724]. Furthermore, the data indicated a main effect of stimulus type [repeated < alternate, *F*_(1, 18)_ = 21.69, *p* < 0.001] and probability [high > low, *F*_(1, 18)_ = 5.77, *p* = 0.027].

Again, an overall analysis revealed a hemisphere-by-probability-by-stimulus interaction, indicating that the influence of perceptual expectation on RS effects varied with hemisphere [*F*_(1, 18)_ = 14.07, *p* = 0.001].

#### PFs

In the left PFs (Figure 2E1), high RS effects could be found in the high-probability context (28% signal decrease, *t* = 6.09, *p* < 0.001), whereas the low-probability context revealed no RS effects (0,4% signal increase, *t* = 0.044, *p* = 0.965). Again, a stimulus-by-probability interaction [*F*_(1, 18)_ = 7.28, *p* = 0.015] superseded the main effect of stimulus type [*F*_(1, 18)_ = 11.29, *p* = 0.003].

RS effects in the right PFs (Figure 2E2) were 14% (*t* = 2.63, *p* = 0.017) and 12% (*t* = 1.86, *p* = 0.079) in the high- and low-probability context, respectively. The data indicated a main effect for stimulus type [*F*_(1, 18)_ = 11.19, *p* = 0.004] and the stimulus-by-probability interaction was not significant [*F*_(1, 18)_ = 0.296, *p* = 0.593]. Different modulations of RS effects by perceptual expectations in the left and right hemisphere was indicated by a significant hemisphere-by-probability-by-stimulus interaction in an additional analysis [*F*_(1, 18)_ = 4.95, *p* = 0.039].

#### Control regions

Although RS effects in the left FFA were more pronounced in the high-probability context than in the low-probability context (21 and 11%, respectively), the data did not reveal a significant stimulus-by-probability interaction [*F*_(1, 18)_ = 3.57, *p* = 0.075] and the main effect of stimulus type denoted common RS effects [repeated < alternate, *F*_(1, 18)_ = 9.86, *p* = 0.006].

In the right FFA, repeated stimuli elicited a decrease in response signal of 14% in both, the high- and low-probability context and thus did not indicate an influence of perceptual expectation [probability-by-stimulus interaction, *F*_(1, 18)_ = 0.112, *p* = 0.752], but rather revealed common RS effects [repeated < alternate, *F*_(1, 18)_ = 12.63, *p* = 0.002]. Activation patterns are depicted in Supplementary Figure A.3.

An overall analysis did not indicate a different modulation of perceptual expectation on RS effects in the left and right hemisphere [hemisphere-by-probability-by-stimulus interaction, *F*_(1, 18)_ = 1.14, *p* = 0.3].

In the left PPA, repeated stimuli caused 12% signal decrease in the high-probability context and 22% signal decrease in the low-probability context. However, the stimulus-by-probability interaction was not significant [*F*_(1, 18)_ = 0.838, *p* = 0.372]. Repeated stimuli generally elicited decreased activation compared to alternate stimuli thus indicating common RS effects [*F*_(1, 18)_ = 20.08, *p* < 0.001].

RS effects in the right PPA were similar in the high- and the low-probability context (12 and 14%, respectively), indicating a main effect of stimulus type [repeated < alternative, *F*_(1, 18)_ = 19.99, *p* < 0.001] and no stimulus-by-probability interaction [*F*_(1, 18)_ = 0.008, *p* = 0.931]. Comparably to the FFA, the hemisphere-by-probability-by-stimulus interaction was not significant [*F*_(1, 18)_ = 0.639, *p* = 0.435]. Results are illustrated in Supplementary Figure A.4.

#### RS modulation between ROIs

Above findings suggest that repetition probability selectively modulates RS effects in object-selective regions. In order to assess this, we conducted an ANOVA including the factors ROI (LOC, FFA, and PPA), probability, stimulus, and hemisphere. The analysis revealed a significant four-way interaction thus showing that RS modulation varies significantly between ROIs [*F*_(1, 18)_ = 18.55, *p* < 0.001]. Given that repetition probability effects were left-lateralized in above results, we conducted two additional ANOVAs (i.e., for the left and right hemisphere) to examine the influence of ROIs in more detail. The findings were in line with our prior analysis, indicating that RS modulation differed between ROIs (probability-by-stimulus-by-ROI interaction) in the left hemisphere [*F*_(1, 18)_ = 11.05, *p* = 0.001] but not in the right hemisphere [*F*_(1, 18)_ = 0.114, *p* = 0.893].

#### Whole brain analysis

**Figure 3:**
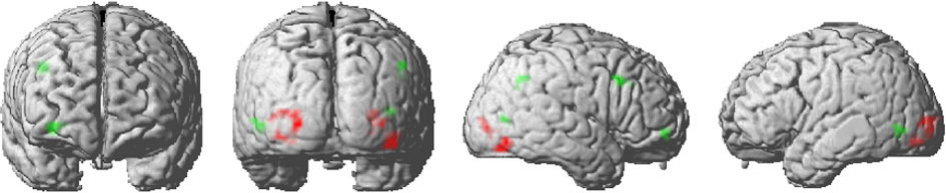
**Activation clusters revealed by the whole brain analyses**. Regions that elicited decreased activation for repeated trials are illustrated in red. Green spots mark clusters where RS effects were modulated by repetition probability (i.e., interaction). All clusters were extracted at a threshold of *p* < 0.001 (uncorrected, with a minimum extent of 10 voxels).

Repeated stimuli exhibited decreased activation compared to new stimuli in the left and right inferior lateral occipital cortex (Figure 3). Further regions indicating decreased activation were localized in the left occipital pole and in the right occipital fusiform gyrus. Notably, there was no significant decrease in activation due to stimulus repetition beyond visual regions that survived a threshold of *p* < 0.001 (uncorrected; 10 voxel extent). We found a significant stimulus-by-probability interaction in bilateral LOC, which exhibited a similar activation pattern as the ROI analyses (*p* < 0.001, uncorrected; 10 voxel extent). A reversed pattern, that is, increased activation for repeated compared to alternative stimuli in the high-probability context, was found in the right middle frontal gyrus and the right frontal pole. The exact coordinates of regions exhibiting a main effect or interactions are listed in Supplementary Table 1.

## Discussion

The objective of the current investigation was to examine whether stimulus repetition probability modulates RS effects during visual object processing in the LOC. According to the PC account, repetition probability modulates the expectation of a repetition, which in turn influences RS effects in object-selective regions. Critically, investigations on perceptual expectation modulations during object processing revealed no influence of repetition probability on RS effects (Kaliukhovich and Vogels, 2011; Kovács et al., 2013), thus challenging the role of top-down effects on RS. To the contrary, the current examination indicates modulatory effects of perceptual expectation on RS during object processing in the LOC. To illustrate, pronounced RS effects could be found in the high-probability context (i.e., 21% signal decrease) whereas activation levels of repeated and alternate stimuli in the low-probability context do not differ in the current investigation.

These findings take issue with the study of Kovács et al. (2013) which indicates that RS effects during object processing in the LO are not modulated by expectations. Of note, we examined RS effects in the whole LOC whereas Kovács et al. (2013) investigated RS effects in the caudal-dorsal subdivision of the LOC, the LO. One could argue that extracting activation from the whole LOC may average across two regions (i.e., the LO and the PFs) which may consist of heterogeneous and functionally divergent subregions also with respect to RS effects (Grill-Spector et al., 1999). Thus, we analyzed the neural response in the two subdivisions of the LOC separately (the coordinates of the LO extracted in the current study correspond to the cluster of Kovács et al., 2013). Notably, both the LO and the PFs indicate a modulation of RS effects by repetition probability in a similar manner as the whole LOC. Thus, undeliberate averaging across functionally divergent regions is not a valid explanation why the current data revealed an effect of perceptual expectations whereas Kovács et al. (2013) failed to find such an effect.

An alternative explanation may be that the diverging extent of repetition probability modulations causes the opposed results. To illustrate, in the study of Kovács et al. (2013), perceptual expectations were induced by 60% repetition probability in the high-probability block and 20% repetition probability in the low-probability block. To the contrary, in the current experiment (and in prior investigations on perceptual expectation as well; e.g., Summerfield et al., 2008, 2011) repetition probability was 75 and 25% in the high- and low-probability context, respectively. To illustrate, the difference in repetition probability between high- and low-probability blocks is 40% in the study of Kovács et al. (2013) compared to 50% in the current investigation. Therefore, the repetition probability manipulation of Kovács et al. (2013) possibly underruns a critical difference which is needed to reliably indicate modulatory effects of perceptual expectation for objects. Furthermore, Kovács et al. (2013) omit reporting analysis of reaction times. Thus, a definite conclusion whether attention or vigilance varied as a function of experimental manipulation is hardly possible by merely interpreting hit rates.

Noteworthy, using the same experimental design, the authors found modulatory effects of perceptual expectation for face stimuli, thus partly excluding different repetition probabilities as a potential explanation. They argue that repetition probability modulation is stronger for face selective neurons as compared to neurons preferring non-face objects. This seems plausible since it has been shown that face processing is a highly specialized mechanism that is capable of even fine-grained differences (Tovée, 1998). A higher sensibility for faces may explain why perceptual expectation modulation on a lower level of repetition probability distinction (40%) becomes visible for face stimuli but not for object stimuli. Vice versa, one may speculate that a greater difference in repetition probability should result in measurable modulatory effects on RS effects during object processing. Critically, this is exactly what we found.

Similar to the study of Kaliukhovich and Vogels (2011), Kovács et al. (2013) could not find effects of repetition probability on RS. However, comparisons between their and the current study must be drawn carefully since the studies differ in a few aspects. First, Kaliukhovich and Vogels (2011) investigated RS effects in a different species (i.e., monkeys). Second, the authors examined electric potentials in the extracellular space, whereas the current study investigated the hemodynamic response succeeding neural activation. However, in a recent electroencephalography study, Summerfield et al. (2011) reported effects of repetition probability. This rules out the possibility that investigating perceptual expectation by means of electric potentials may be less feasible. One possible explanation for the absence of perceptual expectation effects in their study may be that the authors implemented a fixation task. In this task, monkeys were solely rewarded when they remained fixated on a red cross. Accordingly, it seems reasonable to hypothesize that the monkeys attended the fixation cross stronger than the actual stimulus material. This is insofar important, as Larsson and Smith (2012) found that repetition probability modulates RS effects only under conditions of sustained attention toward stimuli. Critically, the modulatory effect of repetition probability vanishes when attention is diverted away. Thus, the selection of an appropriate task may be crucial for the detection of perceptual expectation effects in future studies. Note that differences in species and task cannot (fully) account for the absence of modulatory effects since their results resemble the fMRI investigation on RS effects in humans of Kovács et al. (2013).

As already mentioned, Kovács et al. (2013) and Larsson and Smith (2012) reported influences of top-down expectations on RS effects in the LO. Critically, since perceptual expectation was constantly found for face but not for object stimuli, Kovács et al. (2013) assumed that perceptual expectation effects are dedicated to face processing. Importantly, the current findings demonstrate that perceptual expectation effects on repetition suppression are not restricted to faces. These results are well in line with the PC account, according to which top-down expectations are a general phenomenon in (visual) perception (Summerfield and Egner, 2009). These expectations, which are supposed to feed back informations and to facilitate information processing (Bar et al., 2001, 2006; Bar, 2003), are a core assumption of the PC account.

An interesting finding in the current investigation was that this (apparent) top-down modulation was lateralized. Specifically, RS effects in the left LOC were modulated by repetition probability (thus indicating influences of expectation), whereas the ROIs in the right hemisphere revealed no such effect. As yet, the literature is sparse with regard to the effect of repetition probability on neural activation and much less is known about possible laterality effects in the context of RS. One speculative explanation might be that participants verbally code the stimuli depicted on screen. More specifically, participants may encode their expectation of a forthcoming target in the high-probability context by internally verbalizing the expected object. Accordingly, internal verbalization could account for the left lateralized finding of perceptual expectation effects since this hemisphere is strongly linked with language processes (Vigneau et al., 2006). However, this suggestion is highly speculative and must await more fine-grained investigations in future studies.

Besides our main objective, we investigated whether top-down expectations selectively affect regions that process a current object (i.e., the LOC) or whether expectations influence a broader range of regions in the ventral visual pathway (e.g., the FFA and PPA). Here, the response signal in the FFA and the PPA reveals common RS effects which are not affected by repetition probability. This suggests that top-down expectations reflect a rather specific mechanism which modulated RS effects in the LOC, but not in the FFA and the PPA.

As expected, an exploratory whole brain analysis revealed common RS effects along the ventral visual pathway. In addition, these RS effects varied with stimulus probability in a similar manner as in the region of interest analyses. Interestingly, frontal regions like the right middle frontal gyrus and the right frontal pole revealed the reversed activation pattern with repeated stimuli eliciting higher activation than alternative stimuli [i.e., repetition enhancement (RE)]. Although the co-existence of RS and RE seems challenging, the PC account provides a reasonable framework for this finding. Accordingly, perception requires both, the forming of expectations indexed by repetition enhancement and prediction error signals that indicate the accuracy of the prediction. The previous study by De Gardelle et al. (2013) supports this notion by showing that RE and RS could be found simultaneously, even within the same functional network. Furthermore, RE and RS units could consistently be separated across scanner runs and functional connectivity analyses revealed that RE voxels were relatively more connected to higher visual processing areas whereas RS voxels were connected with lower visual regions. These findings are in line with the current pattern that repeated compared to alternative stimuli revealed decreased activation in the LOC, thus possibly reflecting a decreased prediction error signal, whereas RE effects in higher levels of the processing hierarchy (i.e., frontal regions) might index the development of predictions which then modulate object processing top-down. However, these findings require future investigations (and replications) since the current voxel-based whole brain analysis used an uncorrected threshold and has to be seen as an exploratory analysis. Notably, probability and interaction effects must be interpreted with caution due to the present study design.

Taken together, the present study provides further evidence in favor of the PC model. We found that repetition probability modulates RS effects in the LOC during visual object processing, indicating that the influence of perceptual expectations is not restricted to face perception. These results are consistent with a growing body of studies indicating that the PC model is capable of explaining a broader range of perceptual processes well beyond face processing (e.g., Todorovic et al., 2011; Andics et al., 2013).

### Conflict of interest statement

The authors declare that the research was conducted in the absence of any commercial or financial relationships that could be construed as a potential conflict of interest.
