# Supplementary material for: Neural repetition suppression: evidence for perceptual expectation in object-selective regions
Source: Front Hum Neurosci. 2014 Apr 17;8:225. doi: 10.3389/fnhum.2014.00225 (PMC4029021; doi:10.3389/fnhum.2014.00225)
Supplement: Supplementary file 1 [file DataSheet1.PDF]

Supplementary Material

Neural repetition suppression: evidence for perceptual expectation in object-selective regions.

Lisa Mayrhauser[1*], Jürgen Bergmann[2] , Julia Crone[1,2] & Martin Kronbichler[1,2]

[1] Centre for Neurocognitive Research and Department of Psychology, University of Salzburg, Salzburg, Austria
[2] Paracelsus Medical University, Christian-Doppler Clinic, Salzburg, Austria

***Correspondence**: Lisa Mayrhauser, Centre for Neurocognitive Research, University of Salzburg
Hellbrunnerstraße 34, 5020 Salzburg, Tel: +43-662-8044-5106, Fax: +43-662-4483-3089
Email: lisa.mayrhauser@sbg.ac.at

Region of Interest Analyses

Supplementary Figure A.1 depicts the ROI for the fusiform face area (FFA) as defined by the face > scrambled contrast of the localizer task. **Supplementary Figure 2** represents the ROI for the parahippocampal place area (PPA) (buildings > scrambled).

Supplementary Figure A.3 and **A.4** depict the response pattern of the fusiform face area and the parahippocampal place area, respectively. As expected, neither the FFA nor the PPA reveals modulatory influences of repetition probability since repetition suppression effects are similar in the high- and low-probability context.

Whole brain analysis

Regions that revealed significant activation for the contrasts repeated > alternate, high > low probability and stimulus*probability are listed in **Supplementary Table A.1** Regions were extracted using a threshold of p < .001, uncorrected.

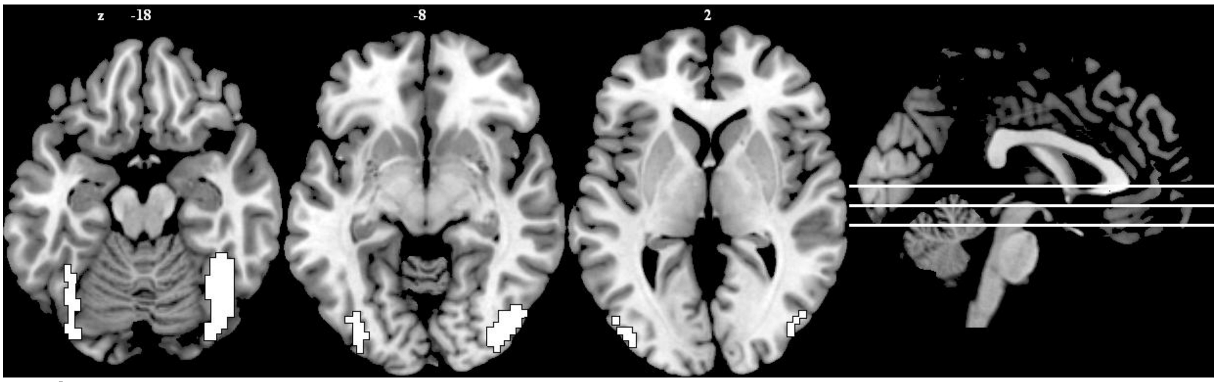

Supplementary Figure A.1

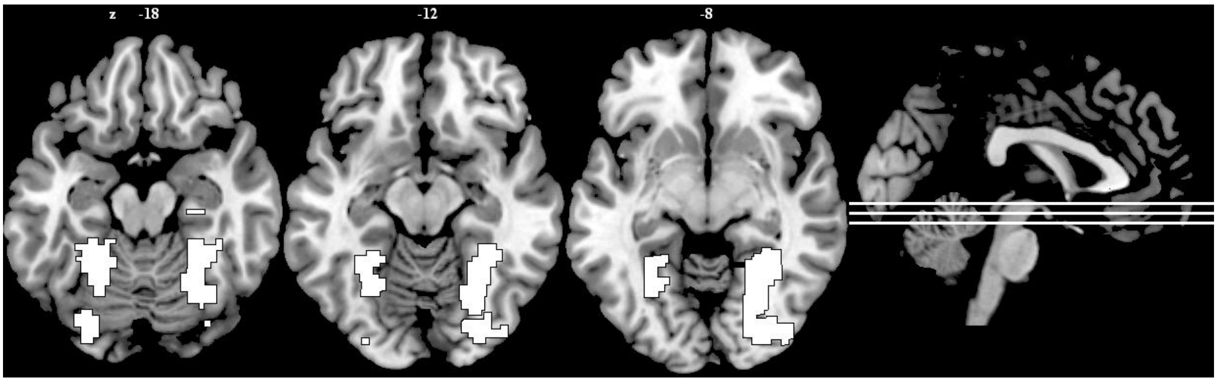

Supplementary Figure A.2

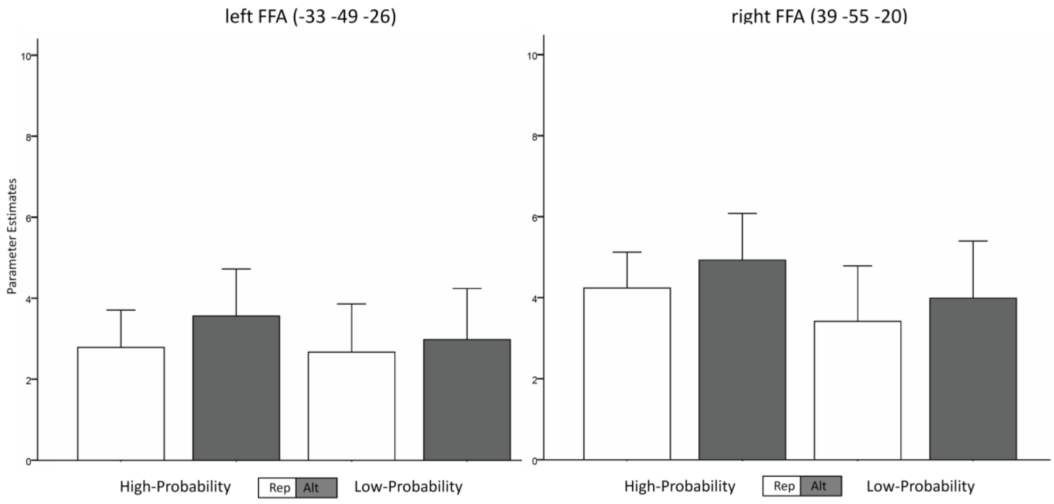

Supplementary Figure A.3

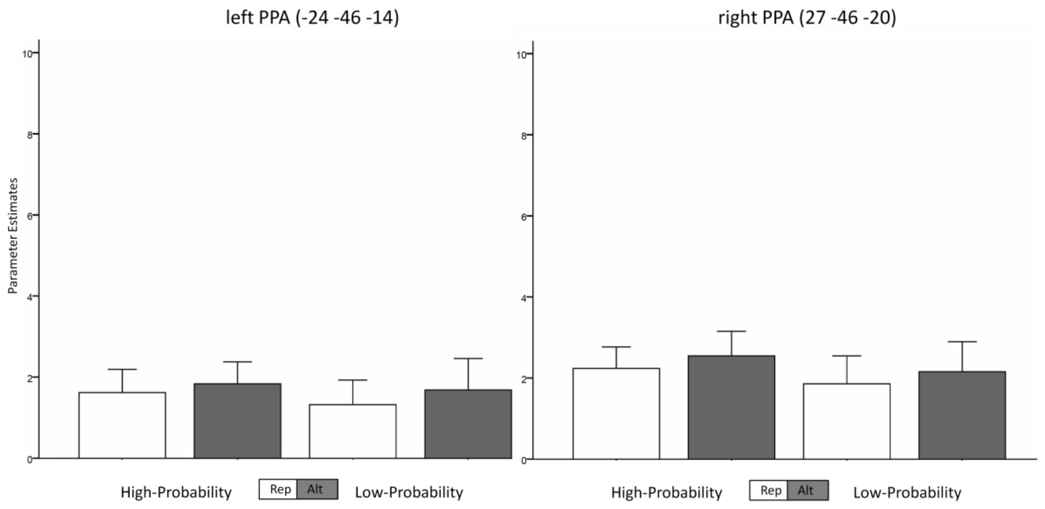

Supplementary Figure A.4

Supplemental Table 1.

	MNI coordinates			Volume	
Region	x	y	z	(voxels)	*Z*
repeated < alternate					
R inferior LOC	42	-70	-11	42	4.08
L inferior LOC	-39	-79	-11	20	3.77
L Occipital Pole	-21	-94	-2	41	4.61
R Occipital Fusiform Gyrus	30	-82	-8	29	3.64
*Stimulus*Probability*					
R LOC	42	-70	7	12	4.13
	51	-55	40	15	3.93
L LOC	-51	-67	-2	16	3.60
R Middle Frontal Gyrus	39	20	37	23	4.37
R Frontal Pole	36	53	-5	16	3.73

Note: threshold of $p < .001$ (uncorrected) with an associated extent threshold (10 voxels).
